# Acute respiratory failure as initial manifestation of conventional osteosarcoma rich in giant cells: a case report

**DOI:** 10.1186/s13256-020-02562-y

**Published:** 2020-11-23

**Authors:** Laura Mosquera-Salas, Nathalia Salazar-Falla, Bladimir Perez, Saveria Sangiovanni, Luz F. Sua, Liliana Fernández-Trujillo

**Affiliations:** 1 General Medicine, Hospitalization Service, Fundación Valle del Lili, Carrera 98 #18-49, 760032 Cali, Colombia; 2 Department of Internal Medicine, Fundación Valle del Lili, Carrera 98 #18-49, 760032 Cali, Colombia; 3 Faculty of Health Sciences, Universidad Icesi, Calle 18 #122-135, 760032 Cali, Colombia; 4 Department of Pathology and Laboratory Medicine, Fundación Valle del Lili, Carrera 98 #18-49, 760032 Cali, Colombia; 5 Clinical Research Center, Fundación Valle del Lili, Carrera 98 # 18-49, 760032 Cali, Colombia; 6 Department of Internal Medicine, Pulmonology Service, Interventional Pulmonology, Fundación Valle del Lili, Carrera 98 #18-49, Tower 6, 4th Floor, 760032 Cali, Colombia

**Keywords:** Osteosarcoma, Giant cells, Mediastinal mass, Ventilatory failure, Airway obstruction, Case report

## Abstract

**Background:**

Osteosarcoma is a malignant tumor of the bone. The giant cell-rich osteosarcoma (GCRO) is a rare histological variant of the conventional osteosarcoma, accounting for 3% of all osteosarcomas. It has a variable clinical presentation, ranging from asymptomatic to multiple pathological fractures, mainly involving long bones, and less frequently the axial skeleton and soft tissues.

**Case presentation:**

We present the case of a 25-year-old Hispanic woman, previously healthy, with a 1-month history of dyspnea on exertion, intermittent dry cough, hyporexia, and intermittent unquantified fever. She presented to the emergency department with a sudden increase in dyspnea during which she quickly entered ventilatory failure and cardiorespiratory arrest with pulseless electrical activity. Resuscitation maneuvers and orotracheal intubation were initiated, but effective ventilation was not achieved despite intubation and she was transferred to the intensive care unit of our institution. The chest radiograph showed a mediastinal mass that occluded and displaced the airway. The chest tomography showed a large mediastinal mass that involved the pleura and vertebral bodies. A thoracoscopic biopsy was performed that documented a conventional giant cell-rich osteosarcoma. The patient was considered to be inoperable due to the size and extent of the tumor and subsequently died.

**Conclusions:**

The giant cell-rich osteosarcoma is a very rare histological variant of conventional osteosarcoma. Few cases of this type of osteosarcoma originating from the spine have been reported in the literature, and to our knowledge none of the reported cases included invasion to the chest cavity with airway compression and fatal acute respiratory failure that was present our case. Radiological and histological features of the GCRO must be taken into account to make a prompt diagnosis.

## Background

Osteosarcoma is the most common primary malignant tumor of the bone following multiple myeloma, constituting 50–70% of all tumors of the skeletal system. It usually presents between 10 and 25 years of age, without gender predilection, although when it affects patients aged ≤ 50 years, the incidence is higher in men. It has a variable clinical presentation, ranging from asymptomatic to local pain, edema, and multiple pathological fractures [1].

Osteosarcoma mainly involves the metaphysis of long bones, such as the femur, tibia, and humerus (91% of cases), and is uncommon in the diaphysis. Less frequently, it involves the maxilla, pelvis, scapula, skull, and vertebrae [2]. When it affects vertebrae, it most frequently invades thoracic vertebrae, followed by lumbar, sacral, and cervical vertebrae in decreasing frequency, presenting usually in the vertebral body or the pedicle and processes. In addition, bone tumors can infiltrate soft tissue near the compromised vertebrae, especially in young people. Compared to other benign and malignant vertebral tumors, osteosarcomas often involve several contiguous vertebral bodies [3].

The giant cell-rich osteosarcoma (GCRO) is a rare histological variant of the conventional osteosarcoma, composed of a combination of mononuclear cells interposed with osteoclast-like giant cells [1]. This variant accounts for approximately 1–3% of all osteosarcomas [1, 4]. Presentation in the thoracic vertebrae is extremely rare, especially when it infiltrates the mediastinum, which can lead to life-threatening complications. Given the rarity of the tumor, we believe physicians would benefit from this report to become familiar with this entity. Furthermore, we emphasize the importance of early diagnosis to provide timely management.

We present the case of a young female patient with a 1-month history of respiratory symptoms, who developed respiratory failure, was diagnosed with an inoperable GCRO, and died.

This report was prepared in accordance with the ethical standards of the institutional ethics committee and with the 1964 Helsinki Declaration. Informed consent was waived by the ethics committee of our institution. Approval was granted by the Ethics Committee in Biomedical Research (IRB/EC No. 382-2016) of the Fundación Valle del Lili to publish this manuscript.

## Case presentation

The patient was a 25-year-old Hispanic woman of African descent. She was the youngest of four siblings, a nursing student, and worked part-time in the administrative department of the Cali City public transportation system. She was previously healthy, was a non-smoker, and did not consume alcohol or other drugs. Her father had arterial hypertension and her mother had type 2 diabetes. She had no other family history. The patient’s medical history included a 1-month history of dyspnea on exertion, intermittent dry cough, hyporexia, and intermittent unquantified fever. She did not seek medical attention for these symptoms until she presented with a sudden increase in dyspnea, at which time she presented to the emergency department of a peripheral institution. Upon admission, she went into acute respiratory failure and cardiorespiratory arrest, with pulseless electrical activity. Despite orotracheal intubation, the patient was difficult to ventilate. She received initial resuscitation maneuvers, was stabilized, and then was transferred to our institution.

Upon arrival at our institution, she was under sedation, with a preserved airway through orotracheal intubation, not lateralized. Her vital signs were: blood pressure, 200/140 mmHg; heart rate, 100 beats per minute; respiratory rate, 16 breaths per minute; temperature, 36 °C; oxygen saturation, 78% despite being on ventilatory support. Physical examination revealed pupils with anisocoria (right 5 mm, left 3 mm) and thick neck with jugular engorgement with no masses. There were no skin lesions. The cardiopulmonary evaluation revealed sinus tachycardia, and on auscultation of the lung, bilateral thick rales were found. There was no collateral circulation, ascites, or masses on the abdomen. Extremities were without edema. The patient was sedated upon admission and continued to be with midazolam and fentanyl drip, plus vecuronium bromide, due to thoracoabdominal uncoupling. The Richmond Agitation and Sedation Scale (RASS) score was 5, making it difficult to evaluate neurological status.

**Fig. 1:**
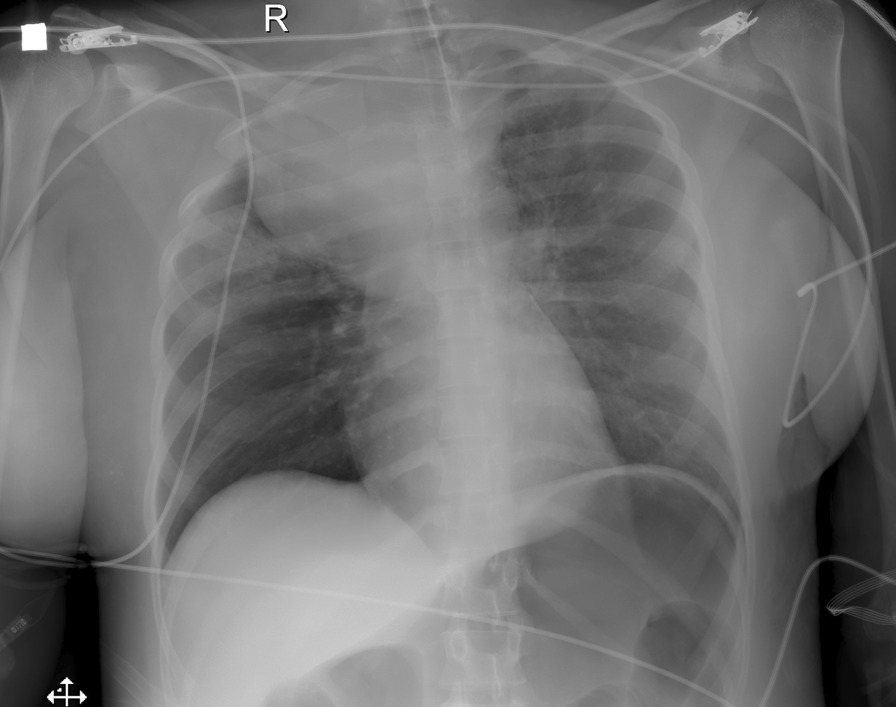
Chest X-ray showing a homogeneous paratracheal superior right mass, displacing the trachea to the left, with a well-defined inferior border. Infiltrates in the right superior lobe, adjacent to the mass. The orotracheal tube was correctly positioned. Other monitoring elements and distension of the gastric chamber are identified

A chest radiography (X-ray) was performed, showing a homogeneous right paratracheal mediastinal mass that compressed and displaced the airway (Fig. 1) to the left. Measures were initiated to stabilize blood pressure, and the conventional orotracheal tube was changed to a ringed one to reduce the collapse due to the extrinsic compression of the tumor. Fiberoptic bronchoscopy was then performed, in which extrinsic compression was observed at the distal end of the orotracheal tube, with no evidence of endotracheal or endobronchial lesions.

**Fig. 2:**
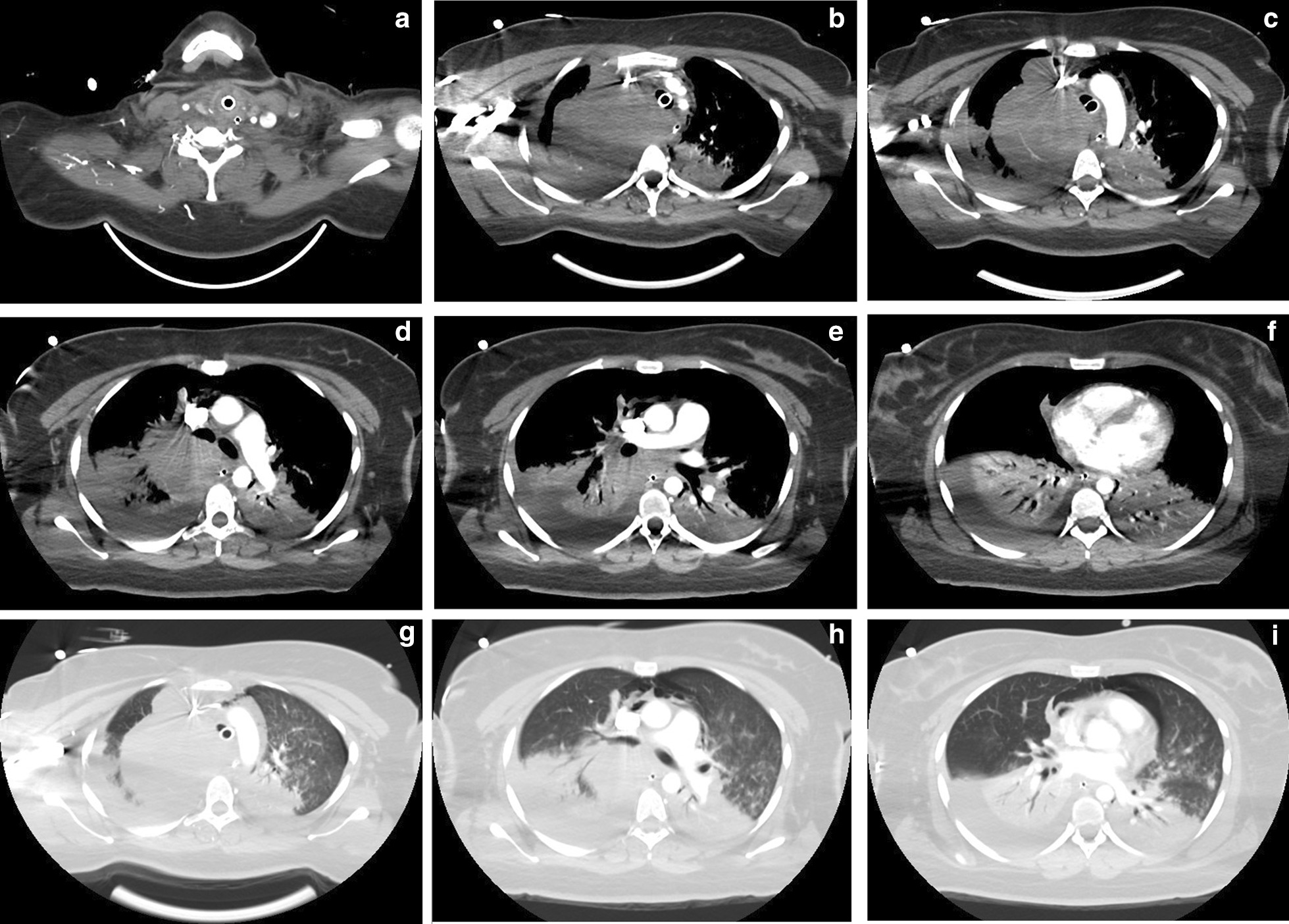
Chest computed tomography scan. **a–c** Sequence of images of the mediastinal window showing a lobulated, homogeneous mass with well-defined borders that originates from the right thoracic operculum, compressing and displacing the trachea and vessels, with infiltrate surrounding it. **d–f** Sequence in which bilateral pleural effusion, predominantly right sided, and bilateral infiltrate in the right lower lobes is observed. **g–i** Lung window displaying the mass, air bronchogram in both lower lobes, pneumomediastinum and bilateral pleural effusion

Evaluations were continued with a chest and neck computerized tomography (CT) scan, revealing a large, lobulated, and solid mass, with well-defined contours, located in the right thoracic operculum that seemed to originate in the pleura and measured 11 × 8.6 × 9.5 cm (L×AP×T). The mass had a nodular component attached to the mediastinal pleura, measuring 27 × 28 mm, which seemed to be thickened. A mass effect of the right pulmonary hilum, right supra-aortic trunks, and superior vena cava were reported. There was a presence of pneumomediastinum and left pneumothorax. Also, lytic bone involvement of the body, pedicle, and transverse processes of the first and second thoracic ( T1 and T2) vertebrae was observed, with a fracture of the bone cortex through the anterior portion of the vertebrae. Additionally, centrilobular opacities formed patches in the superior left lobe and lingula, suggestive of aspiration pneumonitis (Fig. 2).

Blood workup revealed white blood cells 13,47 × 10^3^/μL, neutrophils 85%, hemoglobin 9.90 g/dL, platelets 303 47 × 10^3^/μL, prothrombin time (TP) 19.1 seconds, partial thromboplastin time (TTP) 30.9 seconds, international normalized ratio (IRN) 1.39, blood urea nitrogen 9.50 mg/dL, creatinine 0.80 mg/dL, sodium 137 mmol/L, potassium 4.39 mmol/L, chloride 99.8 mmol/L, lactic acid 2.83 mmol/L, C-reactive protein 4.20 mg/dL, uric acid 5.1 mg/dL, lactic dehydrogenase 592 U/L, calcium 6.58 mg/dL, and phosphorus 3.73 mg/dL. The arterial blood gases examination revealed pH 7.3, partial pressure CO_2_ and O_2_, 44.5 and 122 mmHg, respectively; HCO_3_, 22.3 mmol/L; excess base (BE), − 3.7; SO_2_, 98%. The patient received dexamethasone 8 mg per day and piperacillin/tazobactam 4.5 g every 6 hours.

Both percutaneous puncture and video-assisted thoracoscopy (VATS) were considered for taking a biopsy, but the latter was chosen to guarantee a better quality of tissue sample. In atypical cases such as this one and also taking into account that this case could have corresponded to a hematological malignancy given the age at presentation, being able to analyze the architecture of the tumor from a larger biopsy was vital. The procedure was carried out in the intensive care unit (ICU) without complications.

**Fig. 3:**
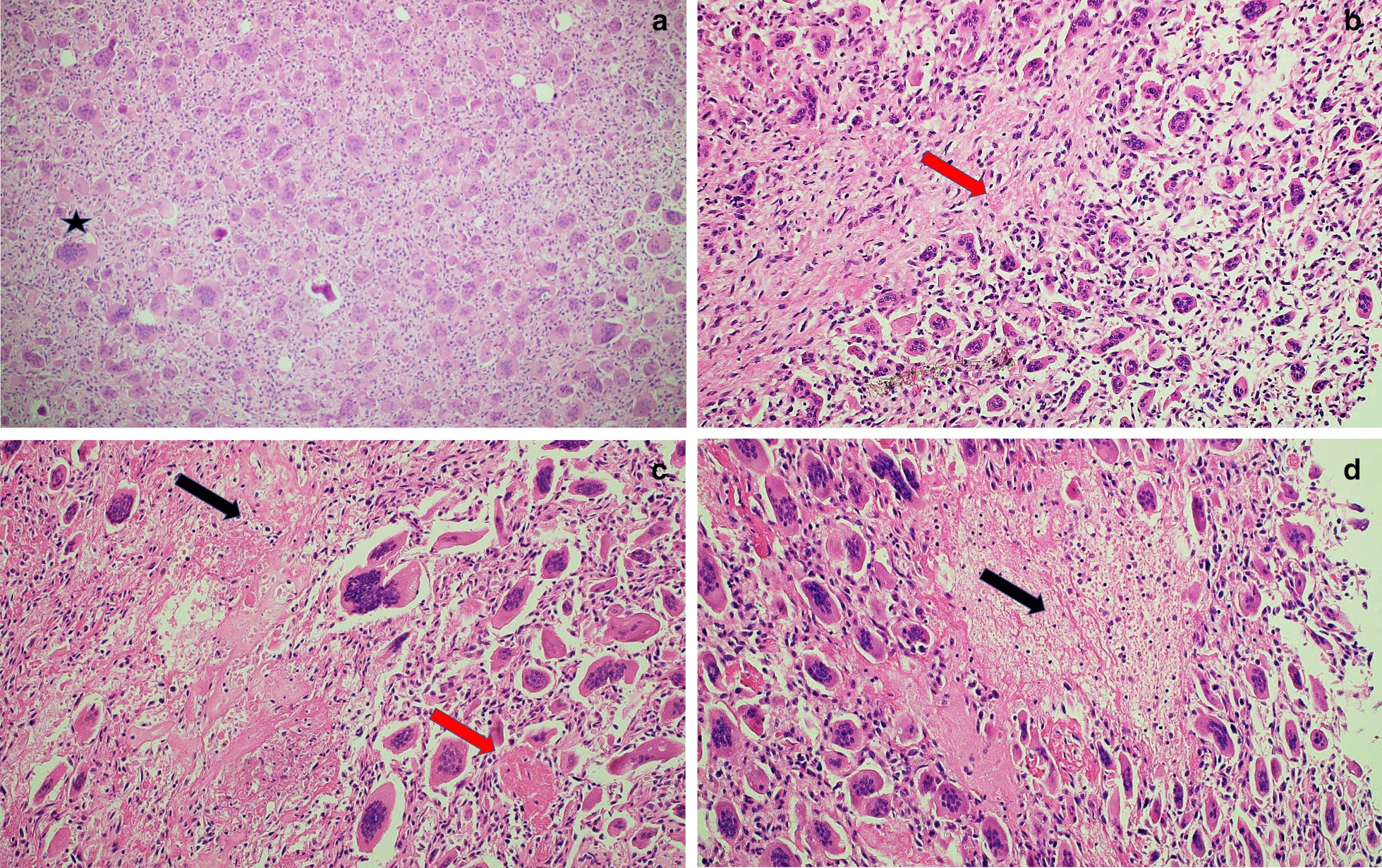
**a** Hematoxylin and eosin staining, ×20. **b**–**d** Hematoxylin and eosin staining, ×40. Neoplastic lesion composed of a mononuclear background and randomly positioned multinucleated giant cells (*star*).* Red arrows* Eosinophilic osteoid, with pleomorphism, in between neoplastic cells,* black arrows* areas of tumoral necrosis

**Fig. 4:**
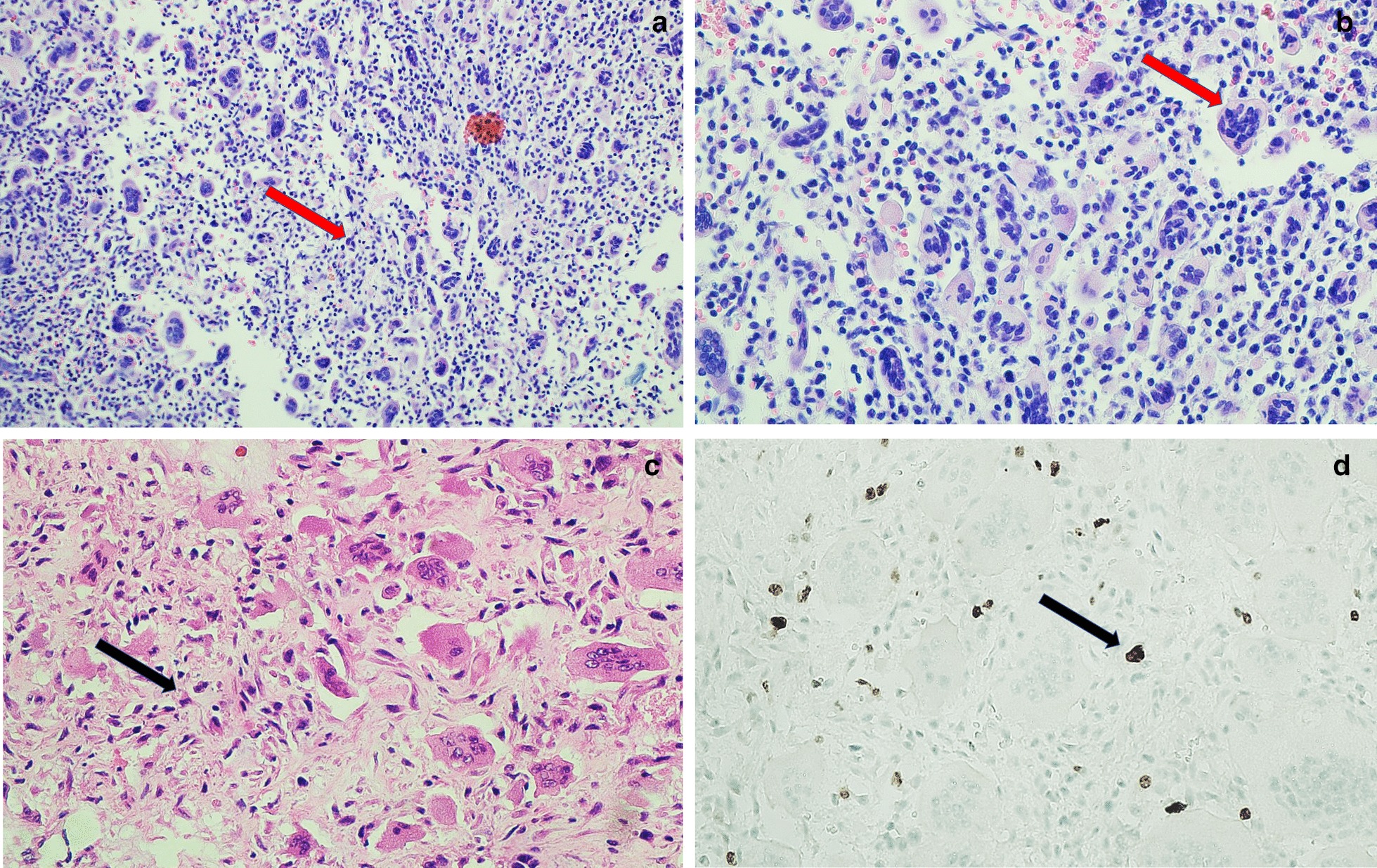
**a** Hematoxylin and eosin staining, ×20. **b**, **c** Hematoxylin and eosin staining, ×40. Neoplastic lesion composed of a mononuclear background and randomly positioned multinucleated giant cells, with marked variability in size and morphology, nuclei without atypia or mitosis (*red arrows*).* Black arrow* Mononuclear cells with cytological atypia and moderate mitosis. **d** Ki-67 proliferation marker, ×40. Black arrow Mononuclear cells with moderate proliferative index

Histologically, a mesenchymal neoplastic lesion was observed with abundant giant osteoclastic cells with round nuclei and mild pleomorphism. The cells were randomly arranged. Similar to stromal tissue, the lesion presented spindle cells with cytological atypia, an increased nuclear-cytoplasmic ratio, and abundant mitosis. Osteoid material was found in isolation, with areas of tumor and ischemic necrosis. No normal bone tissue was observed; the Ki-67 proliferation index was 18%, and the phosphorylated histone H3 (PHH3) proliferation marker was present with three mitoses per high power field (HPF). A diagnosis of GCRO was made (Figs. 3 and 4), with the origin apparently the thoracic vertebrae. Due to clinical compromise and the histological type of the tumor, she was not considered to be a candidate for specific oncological or surgical management. Palliative management continued in the ICU, where comfort was provided to the patient and her family. Five days after admission, she presented with hemodynamic instability, asystole, and died. An autopsy was not performed.

## Discussion

We present the case of a previously healthy young woman with a GCRO that originated from the vertebral bodies and invaded the mediastinum, who presented with respiratory failure and cardiorespiratory arrest as the initial manifestation. To our knowledge, this is the first case report of a GCRO with such characteristics. Although a GCRO is a rare entity, we believe it is important to communicate our experience to raise awareness of this tumor and the importance of detecting it in an early stage.

GCRO is an undifferentiated high-grade sarcoma and is a very rare variant of conventional osteosarcomas. It appears primarily in the distal femur, proximal tibia, and distal tibia. There are few reports of spinal involvement in the literature. The clinical manifestations are nonspecific, with the most common symptoms being pain, a palpable mass, and edema. Symptoms usually last up to 4 months before being diagnosed [3, 4]. Pathological fractures can also present in more advanced stages of the disease, as the tumor affects the cortical bone [1]. In our case, the patient never reported classic local symptoms; since medical care was provided at a very advanced stage of the disease, the clinical presentation progressed rapidly to dyspnea and ventilatory failure secondary to compression of the airway.

Osteosarcomas are diagnosed using imaging techniques, including plain radiography, CT scans, or magnetic resonance imaging [1]. Radiological and histopathological differentiation of GCRO from other benign and malignant giant cell tumors (GCTs) is problematic because the classical radiological appearance of conventional osteosarcomas (such as osteoblastic and osteolytic areas with ill-defined margins, strong periosteal reaction, “Codman’s triangle” due to the elevation of periosteum, and fracture of the bone cortex) are not present, hindering diagnosis [4]. GCRO has been reported in the literature to present with lytic bone destruction with ill-defined margins, typically affecting the diaphysis and metaphysis of long bones, generally without soft tissue involvement and with a weak periosteal reaction [5]. Although CT scans can reveal intraosseous and extraosseous involvement, they has limited value in determining intraarticular growth; for this purpose, magnetic resonance is the better diagnostic option, especially when the aim is to delimit the extent of the tumor, decide upon the best location for the biopsy, and plan the surgical resection. Bone scintigraphy should be used to study bone metastasis. Lactic dehydrogenase (LDH) and alkaline phosphatase (ALP) are typically elevated, although they are nonspecific markers for this disease [1]. In our case, the findings on the CT scan were atypical of GRCO due to the rupture of the bone cortex through the anterior portion of the vertebrae and expansion to the chest, ultimately leading to compression of the airway and respiratory failure. Furthermore, the patient was considered inoperable, so we decided not to perform further imaging. In addition, since the mass compromised the pleura and caused a mass effect on the right pulmonary hilum, right supra-aortic trunks, and superior vena cava, our first diagnosis was a primary tumor of the lung, hence the importance of the histological studies.

Histologically, GRCO presents with numerous giant osteoclast-like cells and a variable amount of osteoid tumor. In conventional osteosarcomas, giant osteoclast-like cells are present in around 13–25% of cases, while GRCO is characterized by these cells covering up the tumor, thus accounting for the name given to this variant. Such cells have large pleomorphic nuclei with an irregular nuclear membrane and a large nucleolus. GRCO also presents with anaplastic and pleomorphic stromal cells and multiple mitotic figures [6]. GRCO must be differentiated from the osteoclastoma or giant cell tumor of the bone (GCTB), a common tumor of the bone, which despite its name does not comprise proliferative osteoclasts cells but mesenchymal stromal osteoprogenitor cells that initiate and maintain osteoclast production instead of osteoblast and osteocyte differentiation [7]. Histologically, GCTB is characterized by large multinucleated giant cells distributed in the stroma of mononuclear spindle cells and monocytes. The monocyte nuclei look similar to those of larger osteoclast-like cells, containing up to 50 nuclei. Furthermore, cystic degeneration, hemorrhage, hemosiderin deposition, sporadic mitotic figures (without atypical mitosis), and increased cell stroma can be observed. The hemosiderin deposition causes the common dark-brown or reddish appearance. This tumor does not produce a matrix-like stroma is the case of GCRO [8].

The histopathological findings for our patient support the description of a GCRO, since abundant osteoclastic giant cells were reported, although with round nuclei and mild pleomorphism, alongside isolated osteoid material and atypical spindle cells. Also, the immunohistochemical study revealed an Ki-67 proliferation index of 18% in one of the highest grade areas. The Ki-67 proliferation index has been reported in the literature to reach up to 30%, perhaps an indication of a tumor’s aggressive potential, but thee is little utility of such a high proliferation index in this type of tumor [9].

GRCOs are treated as conventional osteosarcomas, with surgical resection being the cornerstone of treatment, followed by onchospecific treatment. Since osteosarcomas typically present in long bones, the goal is to perform a limb-saving procedure. Prognosis depends on metastasis, elevated LDH, large tumor size, and poor response to chemotherapy. Radiotherapy is given to patients with positive surgical margins before the initiation of chemotherapy. The most common chemotherapeutic agents used are cisplatin, doxorubicin, ifosfamide, and high-dose methotrexate with leucovorin calcium rescue, or a combination of these drugs. Furthermore, other agents, such as third-generation bisphosphonates, might have an anticancer effect against osteosarcoma cells [1]. Survival rate has been reported to be the same as for conventional osteosarcomas, ranging from 60 to 70% at 5 years, although it decreases to around 20–30% in patients with metastatic disease [1, 5, 8]. Unfortunately, our patient was diagnosed at a very advanced stage of the disease and we were only able to offer palliative care management.

In terms of possible differential diagnosis, physicians must think about causes for central airway obstruction, which can be divided into malignant and non-malignant etiologies. Malignancies that are adjacent to the airway and can cause obstruction by external compression or direct growth of the tumor in the airway, such as thyroid/thymus cancers, and primary mediastinal lymphoma-type tumors are more common in the age group of our patient; less frequently, lung cancer and metastatic extrathoracic malignancies from primary colon, breast, melanoma, and kidney cancers can be found [10]. On the other hand, non-malignant causes are mainly associated with post-intubation or post-tracheostomy stenotic processes, although they have also been associated with tuberculosis infection, granulomatosis with polyangiitis, and transplant-related and idiopathic stenosis. The initial clinical manifestations of these pathologies may be mild, such as cough and dyspnea on exertion, so a high index of suspicion is important for planning exams and treatment. The evaluation includes functional tests, tomographic imaging, and bronchoscopy, and treatment is focused on the cause with interventional pulmonology or chest surgery [11], when appropriate.

## Conclusions

Giant cell-rich osteosarcoma is the least common of osteosarcomas and is difficult to diagnose because its clinical, radiological, and histological characteristics are similar to those of a benign tumor. The involvement of vertebral bodies, as in the case described here, is extremely rare, and to our knowledge, presentation with ventilatory failure due to compression of the airway by the mass has not been reported previously in the literature. The distinctive radiological and histological features of GCRO must be taken into account to reach an opportune diagnosis.

## Data Availability

All data and material are available for sharing upon reasonable request.

## Abbreviations

ALP: Alkaline phosphatase
CT: Computerized tomography
GCRO: Giant cell-rich osteosarcoma
GCT: Giant cell tumor
GCTB: Giant cell tumor of the bone
HPF: High power field
ICU: Intensive care unit
LDH: Lactic dehydrogenase
L×AP×T: Maximum length × anteroposterior length × transversal width
PHH3: Phosphorylated Histone H3
VATS: Video-assisted thoracoscopy
X-ray: Chest radiography
